# Evolution of red blood cell membrane complement regulatory proteins and rheology in septic patients: An exploratory study

**DOI:** 10.3389/fmed.2022.880657

**Published:** 2022-07-28

**Authors:** Julie Vanderelst, Alexandre Rousseau, Nicolas Selvais, Patrick Biston, Karim Zouaoui Boudjeltia, Michaël Piagnerelli

**Affiliations:** ^1^Intensive Care, CHU-Charleroi Marie-Curie, Université libre de Bruxelles, Charleroi, Belgium; ^2^Experimental Medicine Laboratory, CHU-Charleroi Vésale, ULB 222 Unit, Université libre de Bruxelles, Montigny-le-Tilleul, Belgium

**Keywords:** sepsis, red blood cell, complement, deformability, rheology, erythrophagocytosis

## Abstract

**Background:**

During sepsis, red blood cell (RBC) deformability is altered. Persistence of these alterations is associated with poor outcome. Activation of the complement system is enhanced during sepsis and RBCs are protected by membrane surface proteins like CD35, CD55 and CD59. In malaria characterized by severe anemia, a study reported links between the modifications of the expression of these RBCs membrane proteins and erythrophagocytosis. We studied the evolution of RBCs deformability and the expression of RBC membrane surface IgG and regulatory proteins in septic patients.

**Methods:**

By flow cytometry technics, we measured at ICU admission and at day 3–5, the RBC membrane expression of IgG and complement proteins (CD35, 55, 59) in septic patients compared to RBCs from healthy volunteers. Results were expressed in percentage of RBCs positive for the protein. RBC shape was assessed using Pearson's second coefficient of dissymmetry (PCD) on the histogram obtained with a flow cytometer technique. A null value represents a perfect spherical shape. RBC deformability was determined using ektacytometry by the elongation index in relation to the shear stress (0.3–50 Pa) applied to the RBC membrane. A higher elongation index indicates greater RBC deformability.

**Results:**

RBCs from 11 septic patients were compared to RBCs from 21 volunteers. At ICU admission, RBCs from septic patients were significantly more spherical and RBC deformability was significantly lower in septic patients for all shear stress ≥1.93 Pa. These alterations of shape and deformability persists at day 3–5. We observed a significant decrease at ICU admission only in CD35 expression on RBCs from septic patients. This low expression remained at day 3–5.

**Conclusions:**

We observed in RBCs from septic patients a rapid decrease expression of CD35 membrane protein protecting against complement activation. These modifications associated with altered RBC deformability and shape could facilitate erythrophagocytosis, contributing to anemia observed in sepsis. Other studies with a large number of patients and assessment of erythrophagocytosis were needed to confirm these preliminary data.

## Introduction

Alterations of the microcirculation and the cellular metabolism cause multiple organ failure, the final evolution of septic shock leading to death (1–3). In sepsis, alterations of the microcirculation, where the oxygen (O_2_) exchange between blood and cells was performed, and especially their persistence, are associated with morbidity and mortality (2, 4).

Red blood cells (RBCs), as the O_2_ transporters and sensors of local hypoxia, are key elements of the microcirculation (5). These important physiologic functions are related to the RBC membrane integrity which allows the cell to deform and pass through capillaries. Using flow cytometry and ektacytometry, we previously observed altered RBC shape and deformability in septic patients (6–8) and reported that persistence of these alterations was associated with poor outcome (7). We also showed that a more spherical RBC shape in septic patients was associated with decreased sialic acid (SA) content in the RBC membrane (6, 9). These modifications of SA content and sphericity stimulated anaerobic glycolysis as demonstrated by increased intra-erythrocytic concentrations of 2,3 diphosphoglycerate and lactate (9). These results suggest links between the membrane, where glycolytic enzymes are anchored (10), and an enhanced ability of the altered RBCs to produce and liberate ATP by glycolysis (11). Although RBC SA content was decreased, the membrane proteins were not modified (12), in contrast to the lipid part of the RBC membrane in patients with bacterial sepsis (13).

Sepsis is a complex physiopathological model where circulating erythrocytes are vulnerable due, among other things, oxidative injuries occurring under the imbalance of redox homeostasis (14, 15) and activation of the innate immune response with increased complement proteins (16, 17).

RBCs are directly in contact with these complement regulatory proteins and are protected by membrane receptors against membrane damage: CD35, also name Complement Receptor type 1 - CR1, which inhibits complement activation (18) and carries immune complexes or cell fragments to spleen or liver for their clearance from the blood (19). Other RBC complement regulatory proteins included: CD55 (Decay Accelerating Factor - DAF) and CD59 (MembraneAttack Complex Inhibitory Factor – MACIF); both protecting RBCs against hemolysis by activated complement (18). RBC clearance was also enhanced by membrane antibody binding by immunoglobin G (IgG).

Various studies (19, 20) have established a relationship between the modification of the expression of these membrane proteins and the increase in erythrophagocytosis, leading to anemia in various pathologies such as beta-thalassemia and malaria. Nevertheless, none of these studies were performed in sepsis where the complement was also stimulated. In this study, we want to investigate the RBC rheology (shape and deformability) and the expression of different complement regulatory proteins (potential markers of erythrophagocytosis or hemolysis) on the membrane of RBCs from septic patients compared to healthy volunteers.

## Methods

This prospective study was conducted medico -surgical ICU in the CHU-Charleroi, Belgium during 9 months after approval by the local and central ethical committees (ISPPC 008 and CHU-Erasme, Brussels). An informed consent was required for blood analysis.

We enrolled a group of patients with sepsis. Sepsis was diagnosed on the basis of the Third International Consensus Definitions for Sepsis and Septic Shock (21) with a proven bacterial or viral infection.

Patients were enrolled at ICU admission and during days 3–5 if they stayed in the ICU.

Finally, we enrolled a group of healthy hospital employees as controls. Patients and healthy volunteers with RBC pathologies, i.e., sickle cell disease, thalassemia, microcytosis and macrocytosis, were not included in the study.

### Patients

Demographic data were collected from septic patients and volunteers: age, sex, origin of infections, treatment with vasopressors, length of ICU stay and mortality.

For ICU patients, we calculated the Simplified acute physiology score (SAPS 3) (22) and the Sequential organ failure assessment (SOFA) score on day 1 of the ICU admission (23).

We also recorded the following laboratory data: RBC count, hemoglobin (Hb) concentration, hematocrit, erythrocyte count, mean corpuscular volume, leukocyte and platelet counts and lactate and C-reactive protein (CRP) concentrations.

### Flow cytometry and expression of membrane regulatory proteins

The experiments were performed using a flow cytometer: MACSQuant 10 from MiltenyiBiotec^®^.

RBCs (10^6^/mm^3^) were first diluted 500 × in Hanks'Balanced Salt Solution (HBSS). The antibodies (Ab), CD35, CD55, CD59, IgG, were added in the quantities recommended by the supplier BD Pharmigen^®^.

After 15 min of incubation, in the dark and at room temperature, the solution containing the Ab was then washed with 400 μl of HBSS (300 × g for 5 min at room temperature) and then re-suspended in 500 μl of HBSS.

After addition of 1 ml of CD235a to identify RBCs, incubation for 10 min at 4–10°C was performed. For each Ab, we proceeded to the passage of a corresponding iso-typical counting a minimum of 15,000 events.

A compensation matrix was applied to each sample, in order to compensate for the overlapping fluorescence emission spectra of some fluorochromes present on the Ab. Finally, we recorded the percentage of cells expressing fluorescence of the labeled Ab by setting our positivity threshold with the corresponding iso-typical.

#### Measurements of RBC Shape

As change in RBC shape is time dependent, analyzes were completed in a maximum of 90 min. Measurement techniques have been described elsewhere (6, 24). Data were collected on a MacsQuant^®^ Analyzer 10 flow cytometer (Miltenyi Biotec BV, ZZ Leiden, Netherlands). The forward light scatter channels (FSCs) were set on a linear scale. Cell size is the principal component of the FSC signal. For estimation of RBC shape, we used the second coefficient of dissymmetry of Pearson (PCD), applying low shear stresses (12 μl/min RBC flow rate) to allow the RBCs to rotate in the flow without deformation (25). In this technique, we did not add fluorescently labeled agglutinins that can alter RBC shape.

Whole blood (2 μl) was mixed with isotonic (286 mOsm) phosphate-buffer saline at 25°C. This study was limited to 30,000 events and lasted for 15 s. In healthy volunteers, after positioning of the obscuration bar, the cytometer viewed the flow of ellipsoid, biconcave RBCs as essentially two populations of cells, and the FSC histograms showed a typically bimodal distribution of RBCs.

We calculated the PCD = [3 × (mean – median)/ SD] on the histogram as an estimation of the sphericity of the RBCs (6, 24). In general, the PCD values of RBCs in healthy volunteers are around −0.6 and a PCD value of 0 represents a perfect spherical shape.

#### Measurements of RBC deformability

RBC deformability was assessed using ektacytometry (LORRCA; Mechatronics Instruments BV, AN Zwaag, Netherlands). We used the same analytical method as that used by Donadello et al. (7): a suspension of RBCs was mixed with polyvinylpyrrolidone 360 solution, an isotonic viscous medium (PVP, 4%; MW 360 kDa; viscosity 30 ± 2 mPa·s), to obtain a final solution with a constant hematocrit of 0.2%. Using a Couette system composed of a glass cup and a precisely fitting bob, with a gap of 0.36 mm between the cylinders, the liquid solution was sheared and illuminated by a laser beam in order to obtain a diffraction pattern produced by the deformed cells. This diffraction was analyzed by a computer, which also controls the cup rotational speed and the predetermined shear stresses. The elongation index (EI) is calculated as: EI = (*L* – *W*)/(*L* + *W*), where *L* and *W* are the length and width of the diffraction pattern, respectively. The geometry of the diffraction pattern is elliptical. For a given shear stress, the greater the RBC deformability, the higher the EI. At 37°C, we assessed the EI curves for 12 consecutive shear stress values, because human RBC deformability reaches a plateau at 50 Pa: 0.3, 0.48, 0.76, 1.21, 1.93, 3.07, 4.89, 7.78, 12.3, 19.7, 31 and 50 Pa. Interassay variabilities for each shear stress were: 51, 8.2, 3.9, 2.4, 1.7,1.6, 1.1, 1.3, 1.7, 1.2, 1.4 and 1.2%, respectively.

From the shear stress-response curves of shape change, we calculated the maximal RBC elongation (EI max) and curves are presented in logarithmic scales (26).

### Statistical analysis

The laboratory results were anonymized and entered into an Excel spreadsheet. The program used to perform the statistical analysis was SigmaStat version 12.0 (Systat Software Ins, San Jose, California). Results are presented as median values [interquartile range 25%−75%] or percentages and were compared by Mann–Whitney Rank Sum test. Comparisons of EI for SS used the ANOVA test.

The change in PCD or EI over time was evaluated using a Friedman repeat measures analysis of variance with Bonferroni *post-hoc* adjustments. A *p* value < 0.05 was considered as statistically significant.

## Results

### Patients

Nineteen septic ICU patients within 24 h of admission were included and 21 healthy volunteers. Eleveen of the 19 (58%) septic patients were studied during 3–5 days. Clinical characteristics are shown in Table 1. The volunteers were younger than septic patients (53 [34–59] vs. 66 [55–71] years; *p* = 0.01). Fifteen of the 19 (79%) patients with sepsis were in shock at the moment of inclusion.

**Table 1:** Subject demographics, biological characteristics, and outcomes.

	**Healthy volunteers** **(*****n*** **=** **21)**	**Septic patients** **(*****n*** **=** **19)**	***p*** **-Value**
Age (years)	50 [23–57]	65 [55–70]	0.002
Sex (M/F)	10/11	14/5	0.1
SAPS 3	ND	56 [53–69]	
SOFA at day 1	ND	8 [4–10]	
Hemoglobin (g/dl)	14.3 [13.7–16.1]	11.2 [9.3–14.6]	0.017
Hematocrit (%)	43.6 [40.1–47.0]	34.4 [28.3–42.7]	0.012
RBC (10^**9**^/mm^**3**^)	4910 [4322–5380]	3700 [3170–4380]	0.004
MCV (mm^**3**^)	90.9 [88.1–93.6]	94.6 [89.3–96.6]	0.2
WBC (×10^**3**^/mm^**3**^)	6.0 [5.2–6.8]	16.2 [10.3–23.7]	<0.001
Platelets (×10^**3**^/mm^**3**^)	249 [194–308]	258 [234–325]	0.66
Urea (mg/dl)	34 [31–42]	46 [38–42]	0.04
Creatinine (mg/dl)	0.85 [0.78–0.94]	1.23 [0.85–1.75]	0.03
Na^**+**^ (mEq/L)	141 [140–142]	139 [137–141]	0.03
Bilirubin (mg/dl)	0.7 [0.5–0.9]	0.7 [0.4–1.3]	0.8
Glycemia (mg/dl)	82 [77–89]	155 [119–226]	<0.001
CRP (mg/dl)	1 [0.5–4.0]	140 [65–226]	<0.001
Lactate (mmol/L)	ND	1.6 [1.3–2.1]	
pH	ND	7.41 [7.38–7.44]	
PaO_**2**_/FiO_**2**_	ND	203 [151–290]	
Vasopressor dosesmcg/kg h (*n* = 9)	ND	0.17 [0.09–0.23]	
ICU length of stay (days)	ND	5 [4–6]	
ICU Mortality (%)	ND	6 (32)	

*Results are presented as median [25th−75th] percentiles and compared by Mann–Whitney test. NR, non-relevant; SAPS III, Simplified acute physiology score 3; and SOFA, sequential organ failure assessment; RBC, red blood cell; MCV, mean corpuscular volume; WBC, white blood cell; CRP, C-reactive protein*.

Sepsis was due to peritonitis in eight patients (42%); pneumonia in four (21%); urinary tract infection in two (10%); meningitidis in two (10%) and osteitis in one (5%). Three of these patients (16%) also had a positive blood culture at day 1. ICU mortality was 32%.

### Biological data

ICU patients at admission were significantly more anemic than volunteers (RBC count for septic patients: 3,700·10^6^/mm^3^ vs. for healthy volunteers: 4,910·10^6^/mm^3^; *p* = 0.004; Table 1). As expected, inflammation suggested by WBC count and CRP concentrations were significantly higher in septic patients (WBC count for septic: 16.2.10^3^/mm^3^ vs. for healthy volunteers: 6.0.10^6^/mm^3^; *p* < 0.001). There was also more acute kidney failure in septic patients (creatinine for septic patients: 1.23 [0.85–1.75] vs. 0.85 [0.78–0.94] mg/dl for healthy volunteers, *p* = 0.032; Table 1).

### Flow cytometry and expression of membrane regulatory proteins

At ICU admission, we noted a significant decrease only in the expression of CD35 on the RBC membrane surface in septic patients compared to volunteers (Table 2). In the 11 septic patients studied for 3–5 days, no significant changes in membrane protein expression over time were observed (Table 3).

**Table 2:** Comparisons of membrane receptor proteins and Ig G at ICU admission.

	**Healthy volunteers** **(*****n*** **=** **21)**	**Septic patients** **(*****n*** **=** **19)**	***p*** **-Value**
CD 35	23.04 [11.39–32.57]	12.30 [9.02–22.92]	0.042
CD 55	95.82 [94.93–97.96]	95.40 [94.30–96.20]	0.78
CD 59	92.34 [86.40–93.61]	91.31 [80.22–94.88]	0.11
Ig G	1.55 [0.90–2.35]	1.29 [1.03–2.18]	0.87

**Table 3:** Evolution of the receptor membrane receptor proteins and Ig G in septic patients at day 1 and 3–5 (*n* = 11).

	**Day 1** **(*****n*** **=** **11)**	**Day 3–5** **(*****n*** **=** **11)**	***p*** **-Value**
CD 35	11.30 [7.80–25.00]	15.60 [8.50–20.30]	0.56
CD 55	94.97 [93.15–96.12]	94.35 [91.65–95.28]	0.46
CD 59	82.43 [69.95–93.45]	92.62 [83.21–93.34]	0.11
Ig G	1.69 [1.28–2.48]	1.76 [1.11–6.2]	0.64

#### RBC shape

**Figure 1:**
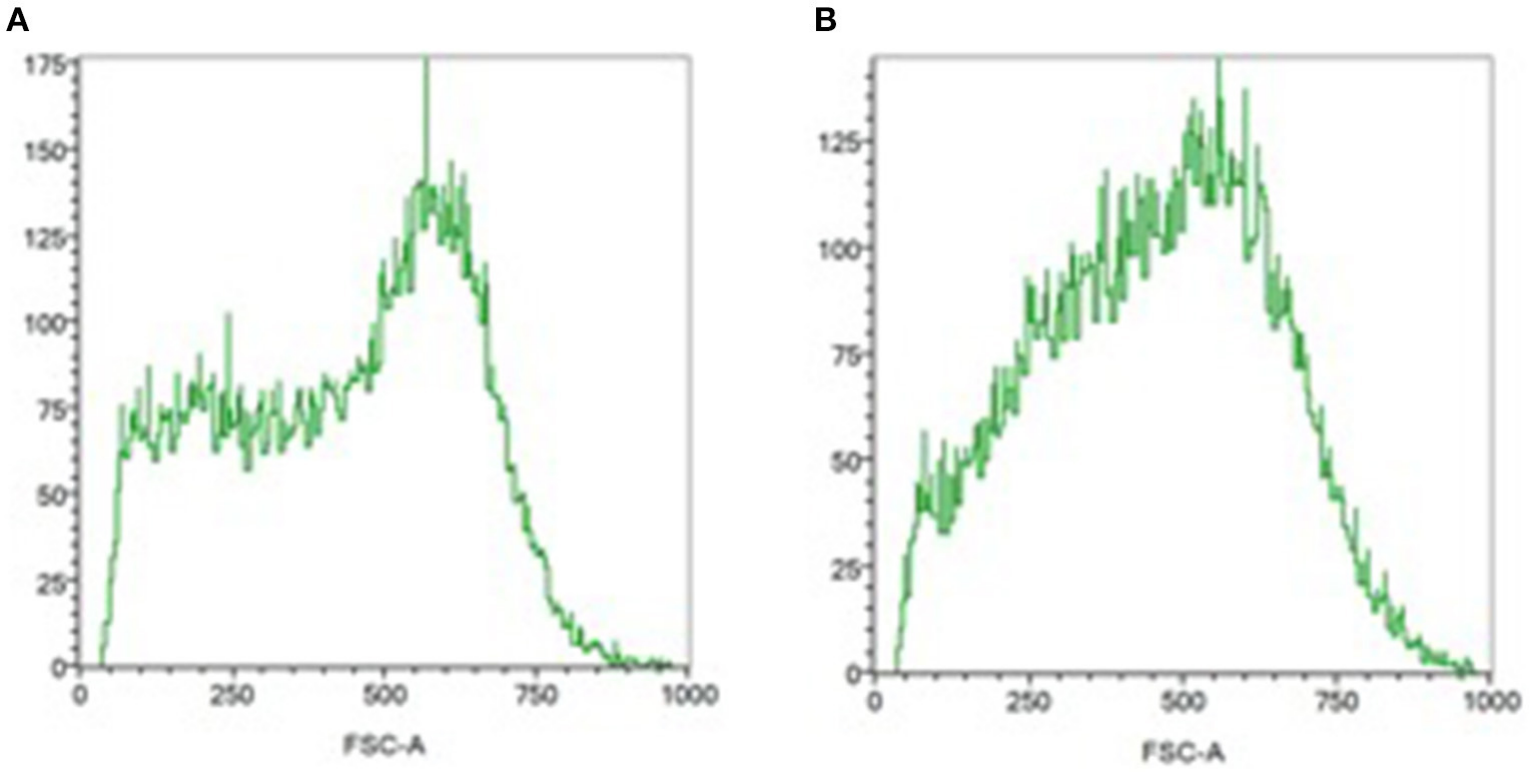
Assessment of the RBC shape by flow cytometry. RBC distribution histograms relating number of events and forward light scatter (FSC) distribution (size estimation). **(A)** In normal biconcave, ellipsoid red blood cells, the FSC distribution (size estimation) is bimodal. **(B)** Monomodal distribution is observed in septic patient, suggesting a more spherical shape.

At ICU admission, the PCD was significantly lower in septic patients than in healthy volunteers suggesting a more spherical shape (−0.399 [−0.563; −0.180] vs. −0.538 [−0.657; −0.491]; *p* = 0.026; Figure 1). The PCD remained lower on day 3–5 in 11 septic patients: for ICU admission −0.398 [−0.560; −0.075] vs. −0.404 [−0.516; −0.129] on day 3–5; *p* = 0.21.

#### RBC deformability

**Figure 2:**
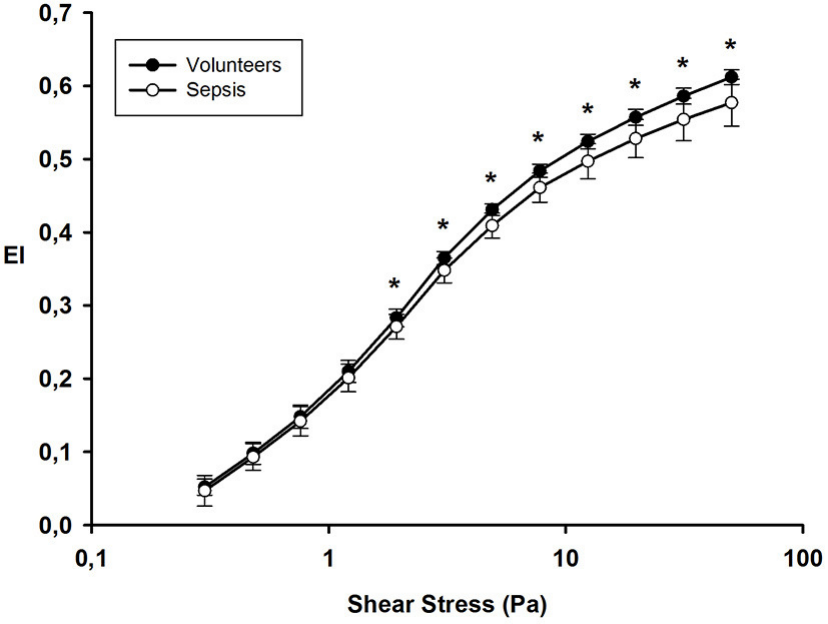
Elongation index at different shear stress values on day 1 in healthy volunteers and septic patients. *p value <0.05.

At ICU admission, deformability was significantly impaired in RBCs from septic patients compared to RBCs from healthy volunteers for shear stress ≥1.93 Pa (Figure 2). The EI max for septic patients was 0.577 ± 0.032 compared to 0.612 ± 0.010 for healthy volunteers; *p* < 0.001.

We followed 11 septic patients until day 3–5. The EI max on day 1 (0.576 ± 0.041) was similar to day 3–5 (0.586 ± 0.02); *p* = 0.25.

## Discussion

Sepsis is a complex disease characterized by inflammation, altered microcirculation and mitochondrial dysfunction (1, 2). Enhanced activation of the complement contributes to the inflammation and leads to cells lysis and phagocytosis by the reticuloendothelial cells (16, 17).

RBCs, as the oxygen transporters and sensors of local hypoxia are in contact with the complement proteins and are protects by several complement regulatory proteins. RBC membrane is the key element of deformability (5) and it's modifications could alter the RBC shape and rheology.

In this study, we observed a more RBC spherical shape and altered deformability in septic patients already at ICU admission. These alterations persisted at day 3–5. On these RBCs from septic patients, only membrane CD35 expression significantly decreased during the first 5 days.

Previously, we observed altered RBC shape using flow cytometry and RBC deformability using ektacytometry in septic patients (6, 8, 27) and reported that persistence of these alterations was associated with poor outcome (7). In this work, we confirmed these results on the shape and deformability of RBCs. These alterations may be secondary to changes in the membrane, which is the key element in the RBC deformability (6). For example, we showed that a more spherical RBC shape in septic patients was associated with decreased sialic acid content – a carbohydrate- on the RBC membrane (9) and we observed an increased neuraminidase activity, an enzyme that cleaves sialic acid on the RBC membrane in these septic patients (9).

The complement system represents the first line of defense that is involved in the clearance of pathogens, dying cells and immune complexes via opsonization, induction of an inflammatory response and the formation of a lytic pore. RBCs, as a circulating blood cells, are in contact with enhanced complement proteins observed in sepsis and expresses membrane complement regulatory proteins to limit complement activation. Decreased expression of the membrane regulatory proteins may result in complement activation and accelerated removal of these modified RBCs.

CD 35 also called CR1, is a surface protein that binds C3b in circulating immune complexes, making these complexes available for uptake by macrophages of the reticuloendothelial system (18).

Complement proteins deposits were already observed in RBCs from ICU patients. Indeed, Muroya et al. observed significantly higher amounts of C4d on the RBCs surface from 40 trauma patients compared to RBCs from 17 healthy volunteers (28). *Ex-vivo* addition of RBCs from universal donors with sera from trauma patients promotes C4d depositions and limited their deformability assessed by microchannel arrays. Modifications of band 3 phosphorylation, accelerated calcium influx and enhanced nitric oxide production could be the origin of altered deformability (29).

Recently, Lam et al. (30) compared complement (C3b and C4b) deposition on RBCs in COVID-19 patients compared to non-COVID-19 septic patients and healthy volunteers. These authors observed on 11 septic patients an increase of RBCs with bound C3b/iC3b/C3dg and C4d but also in COVID-19 patients compared with healthy volunteers. These results agreed with our results and suggested complement activation products were present on the RBC membrane from septic patients. Kisserli et al. (31) also observed an acquired decrease of RBC CR1 density from COVID-19 patients. They observed a relationship between decrease RBC CR1 density and severity of hypoxemia and in 32 patients with a longitudinal study, they observed a decrease of RBC CR1 density at day 10. These results were difficult to extrapolate in septic non-COVID 19 patients due to particular pathophysiology of the SARS-CoV 2 disease (32). Moreover, Kisserli et al. (31) compared these results to RBCs from healthy volunteers but not septic patients.

Waitumbi et al. (19) observed deficiencies in CR1 but also in CD 55 and an increase in CD 59 in RBCs from patients with severe anemia due to *Plasmodium falciparum*. These results suggested an increase in erythrophagocytosis in this pathology. We found the same results in our septic patients but only for CD35 and not for the other complement regulatory proteins like CD 55 and CD 59, no IgG deposit on the RBC membrane. The difference with our results may be explained by the number of patients studied and difference in the characteristics of the included patients (children with anemia due to malaria in the study of Waitumbi et al. without sepsis and adult ICU septic in our study). Age of the subjects studied was probably important. Indeed, Waitumbi et al. (33) observed a lower RBC complement regulatory protein in young children and increased into adulthood. In contrast, we included only adult patients in our study. Moreover, CD 55 and CD 59 protects RBCs against hemolysis by the complement and this phenomenon was not frequently observed in sepsis.

Mechanisms by macrophages of the reticuloendothelial system recognize senescent RBCs for clearance include enzymatic dysfunctions, neoantigens on RBC surface, exposure phosphatidylserine and decreased deformability due to membrane damage (34).

Our study suggests that a decrease in CD35 expression on the RBC surface may be part of the accelerated aging process and erythrophagocytosis in RBCs from septic patients as well as an increase in RBC sphericity. Blocking complement activation could therefore be an interesting therapy to limit the development of anemia in these patients, and possibly tissue damage related to complement proteins activation, as suggested in COVID-19 patients (19, 35). Moreover, a selective inhibition of one complement protein rather than the whole complement activation pathway would be more interesting in terms of morbidity, especially against the increased risk nosocomial infections (36). Nevertheless, further studies on a large number of non-COVID-19 sepsis or septic shock patients are needed to confirm this hypothesis.

There are several limitations to our study. First, the number of subjects included in the study is limited. Due to ICU discharges or deaths, our sample has been further reduced over time. Therefore, a study with a larger population and longer than 5 days is needed to confirm the results obtained.

Second, we have voluntarily limited ourselves to the observation of the expression of three membrane proteins (CD 35, CD 55, CD 59) and IgG. It would also be interesting to study the expression of RBC membrane CD 47 because it plays a role in the phagocytosis process (37). This could reinforce our hypothesis of accelerated RBC aging in sepsis. Third, we observed a decrease of RBC membrane regulatory proteins but we have not measured blood complement proteins to confirm enhancement due to sepsis. Four, we did not included a group of non-septic ICU patients. Those patients with moderate inflammation may also have alterations on RBC membrane complement regulatory proteins. Indeed, we have previously observed an altered RBC shape in these patients (6).

In conclusion, our results show that the RBCs of septic patients have an increased sphericity as well as decreased deformability, and this pattern did not change over 3–5 days. In addition, we observed a decrease in the expression of membrane complement regulatory protein CD 35. These shape, deformability and membrane changes observed on the RBCs of septic patients could promote erythrophagocytosis.

## Data availability statement

The raw data supporting the conclusions of this article will be made available by the authors, without undue reservation.

## Ethics statement

The studies involving human participants were reviewed and approved by ISPPC 008 and CHU-Erasme, Brussels. The patients/participants provided their written informed consent to participate in this study.

## Author contributions

JV, NS, KZ, and MP designed the study. JV, AR, NS, PB, KZ, and MP performed the research. JV, KZ, and MP wrote the paper. All authors revised the paper critically and approved the final version.

## Conflict of interest

The authors declare that the research was conducted in the absence of any commercial or financial relationships that could be construed as a potential conflict of interest.

## Publisher's note

All claims expressed in this article are solely those of the authors and do not necessarily represent those of their affiliated organizations, or those of the publisher, the editors and the reviewers. Any product that may be evaluated in this article, or claim that may be made by its manufacturer, is not guaranteed or endorsed by the publisher.
